# Off-pump versus on-pump coronary artery bypass graft surgery outcomes in patients with severe left ventricle dysfunction: inverse probability weighted study

**DOI:** 10.1186/s12872-022-02895-0

**Published:** 2022-11-17

**Authors:** Ali Sheikhy, Aida Fallahzadeh, Khalil Forouzannia, Mina Pashang, Masih Tajdini, Shahram Momtahen, Soheil Mansourian, Mahmoud Shirzad, Saeed Sadeghian, Kaveh Hosseini

**Affiliations:** 1 Research Department, Tehran Heart Center, Tehran University of Medical Sciences, Tehran, Iran; 2 Non-Communicable Disease Research Center, Endocrinology and Metabolism Population Sciences Institute, Tehran University of Medical Sciences, Tehran, Iran; 3 Department of Surgery, Tehran Heart Center, Tehran University of Medical Sciences, Tehran, Iran; 4 Tehran Heart Center, Tehran University of Medical Sciences, Tehran, Iran; 5 Department of Cardiology, Tehran Heart Center, Tehran University of Medical Sciences, North Karegar Ave, PO Box: 1411713138, Tehran, Iran

**Keywords:** Coronary artery bypass surgery, Ejection fraction, Left ventricular dysfunction

## Abstract

**Objective:**

In this study we aimed to compare on-pump and off-pump coronary artery bypass grafting (CABG) outcomes in patients presented with low left ventricular ejection fraction (EF) as a high-risk group of patients.

**Methods:**

In this registry-based study from 2014 and 2016, all patients with severe left ventricular dysfunction (EF less than 35%) were included and followed until 2020. The median follow-up period was 47.83 [38.41, 55.19] months. Off pump CABG (OPCABG) was compared with on-pump CABG (ONCABG) in terms of mid-term non-fatal cardiovascular events (CVEs) and all-cause mortality. Propensity score method (with inverse probability weighting technique) was used to compare these two groups.

**Results:**

From 14,237 patients who underwent isolated CABG, 2055 patients with EF ≤ 35% were included; 1705 in ONCABG and 350 patients in OPCABG groups. Although OPCABG was associated with lower risk of 30-days mortality (Odds Ratio [OR]: 0.021; Confidence Interval [CI] 95% [0.01, 0.05], *P* < 0.001); there was no significant difference between OPCABG and ONCABG in term of mid-term mortality and non-fatal CVEs ((Hazard ratio [HR]: 0.822; 95%CI [0.605, 1.112], *p* = 0.208) and (HR: 1.246; 95%CI [0.805, 1.929], *p* = 0.324), respectively). Patients with more than three traditional coronary artery disease risk factors, had more favorable outcomes (in terms of mid-term mortality) if underwent OPCABG (HR: 0.420; 95%CI [0.178, 0.992], *p* = 0.048).

**Conclusion:**

OPCABG was associated with lower risk of 30-days mortality; however, mid-term outcomes were comparable in both OPCABG and ONCABG techniques.

**Supplementary Information:**

The online version contains supplementary material available at 10.1186/s12872-022-02895-0.

## Introduction

Coronary artery bypass grafting (CABG) has long been used for surgical revascularization in patients with coronary artery disease (CAD) and is most commonly performed using cardiopulmonary bypass (CPB) (on-pump CABG, ONCABG) [1]. Besides other known predictive factors such as diabetes mellitus, kidney disease and advanced age; left ventricular dysfunction is an important risk factor that might affect surgical coronary revascularization outcomes [2, 3]. It has been shown that reduced ejection fraction (EF) is associated with poor short- and long-term outcomes after CABG and ejection fraction is a component of preoperative risk assessment [4, 5]. Moreover, patients with reduced EF have several comorbid conditions that may affect the post-CABG outcomes [6]. However, CABG is the treatment of choice in those with low left ventricular EF and several studies have shown a long-term survival benefit after CABG in those patients, compared to medical treatment [6]. CABG can be done with two techniques; off vs. on pump and although, several studies compared these methods, there are too many controversies in results [7]. As technology has advanced, OPCABG is well tolerated by most patients [8]; however, this procedure may be associated with incomplete revascularization and also hemodynamic deterioration especially in patients with left ventricular dysfunction [9, 10]. Multiple reports have been published on the outcomes of ONCABG and OPCABG in patients with low left ventricular EF, however the results are conflicting [11, 12].

In this study, we aimed to compare early and mid-term outcomes of ONCABG vs. OPCABG in patients presented with left ventricular dysfunction.

## Methods

### Study cohort

This study is a registry-based cohort which conducted retrospectively in clinical registry of Tehran Heart Center [13]. We reported this study according to the Strengthening the Reporting of Observational studies in Epidemiology (STROBE) statement. Patients who underwent isolated CABG from January 2014 to December 2016 were retrospectively evaluated, additionally patients with lack of adequate data were excluded from the study. Inclusion criteria comprised patients with: (1) A pre-operation EF ≤ 35%, which was evaluated by transthoracic echocardiography (TTE); (2) Surgical revascularization criteria for ischemic heart disease [14]; and (3) No requirement for concomitant valve surgery or minimally invasive direct coronary artery bypass‐surgery. The main exclusion criteria were incomplete registry data and loss to follow up. Conclusively, 2055 patients were selected for this study.

### Definition of variables

EF was evaluated through an expert cardiologist via eyeballing and Simpson technique. Diabetes mellitus was defined as fasting plasma glucose ≥ 126 mg/dL and/or random plasma glucose ≥ 200 mg/dL and/or hemoglobin A1c (HbA1c) ≥ 6.5% [15] and/or treatment with either oral hypoglycemic agents or insulin. Minimum systolic blood pressure of 140 mm Hg or a minimum diastolic blood pressure of 90 mm Hg or a history of antihypertensive therapy labeled as hypertension. Dyslipidemia considers as existence one of follows, minimum total cholesterol level of 240 mg/dL; LDL-C level more than 160 mg/dL; a minimum triglyceride level of 200 mg/dL; and HDL-C of less than 40 mg/dL in men and less than 50 mg/dL in women; or a history of prescribed lipid medications based on the National Cholesterol Education Program (NCEP) Adult Treatment Plan (ATP) III [16]. Renal failure was defined as glomerular filtration rate < 60 mL/min/1.73 m^2^ or stage 3a and higher chronic kidney disease. Cerebrovascular accident was defined as, ischemic stroke, hemorrhagic stroke, and transient ischemic attack. A family history of CAD was defined as having a first-degree relative with a history of CAD; i.e., acute myocardial infarction or documented CAD, which diagnosed by either invasive coronary angiography or computed tomography coronary angiography. Cigarette smoking status was defined as current smoker and stated from the patient’s self-reported status. Opium consumption was defined as the current consumption of opium either smoking opium or drinking opium dissolved in tea. Patients divided into three categories in the term of urgency of the procedure, emergent (surgery should take place as soon as possible, in the following 6 h), urgent (surgery should take place in the following 6–24 h), and elective.

### Surgical technique

To reduce the effect of differential expertise bias, all surgeons who performed procedures were highly experienced in both OPCABG and ONCABG. All surgeons which included in this study has been performed at least 500 OPCABG and ONCABG procedures. The selection of patients to receive either on-pump or off-pump CABG was by surgeon discretion at the time of the procedure. “No-touch” technique was preformed to harvest saphenous vein grafts (SVG) and “pedicled technique” was performed to harvest left and right internal mammary arteries (LIMA and RIMA). The procedure routine was using LIMA for the left ascending artery (LAD) and SVG for right coronary, left circumflex, and diagonal artery, furthermore the choices of using grafting conduits was based on surgeon’s preference concerning.

For ONCABG procedure, single right atrium and aortic cannulation was made to achieve CPB, furthermore, Heparin was used to conserve activated clotting time (ACT) at ≥ 480 s. During the surgery anterograde cold blood cardioplegia was made. Protamine sulfate prescribed to neutralize the Heparin at the end of surgery. The patients’ systemic temperature was sustained at 36 °C to avoid hypothermia-induced arrhythmia.

For OPCABG procedure, carbon dioxide blower (Medtronic Inc., Minneapolis, MN) was used for better visualization of operative field and anastomosis cites. Heparin was given to reach ACT ≥ 350 s. Proximal anastomoses to the aorta was made by 6‐0 monofilament sutures, while 8–0 monofilament sutures was used for distal anastomosis.

### Follow up and study endpoints

The patients follow up protocol was as follows; 4, 6, and 12 months after surgery and annually after last visit through attending visits at the post-op clinics. For individuals who were incapable to appear at the clinics, telephone interviews were made.

The primary endpoints were in-hospital mortality (which was defined as death occurring during the same hospital admission or first 30 days mortality after procedure), mid-term all-cause mortality, and mid-term non-fatal cardiovascular events (CVEs) occurrence (acute coronary syndrome, need for repeat revascularization [percutaneous coronary intervention [PCI] or redo-CABG], stroke or transient ischemic attack).

### Statistical analysis

Descriptive statistics were used to describe baseline characteristics, subsequently, categorical variables were described as absolute and frequencies, and continuous variables were reported as mean and standard deviation or median and interquartile range according to their distribution. The Fisher’s exact test or the chi-squared test was used to compare categorical variables. Normally and non-normally distributed continuous variables were compared using Student t-test and Mann–Whitney U test, respectively.

Inverse probability weights (IPW) used to stabilize potential selection biases of treatment, balance variables, and confounders adjustment (Additional file 1: Table S1). Weights were calculated from propensity score (PS) (Additional file 1: Figure S2), which was generated by predicted probabilities of logistic regression on identified potential confounders. The C-statistic for the model was 0.81 (Additional file 1: Figure S1). Weights for each case (W_i_) calculated as 1/PS(X_i_) for Off-pump surgery, and 1/(1 − PS(X_i_)) for On-pump surgery. The confounders selected based on three main criteria. First of all, we considered only available variables in our data bank. Second, we considered clinically proven confounders for IPW estimation. In the last step we used bidirectional selection, by utilizing multivariable cox-regression to include other variables with *P*-value < 0.25 in our estimation.

Sensitivity analysis conducted using multivariable proportional hazard models. The standardized mean difference (SMD) used as balance metric to evaluate the difference between distributions of a pre-treatment variable, balance indicator considered as ‘SMD < 0.1’(Additional file 1; Table S2).

Event rates were based on Kaplan–Meier estimates in time to first event. log-rank test and univariate proportional hazard model were preformed to compare to surgical methods. On-pump surgery was considered as reference in all reported hazard ratios (HRs). The multiple comparisons of off-pump strategy effect in the subgroup analysis were performed using multiple tests with Bonferroni-adjusted correction and (*p*-value < 0.007) considered as significant.

All statistical analyses were conducted applying IBM SPSS Statistics for Mac, version 26.0 (Armonk, NY: IBM Corp.) and R version 4.0.3. Besides, we used several packages in R: "survival" (package for survival analysis in R), "survminer" (drawing survival curves), and “ggplot2”.

## Results

### Study population

**Fig. 1:**
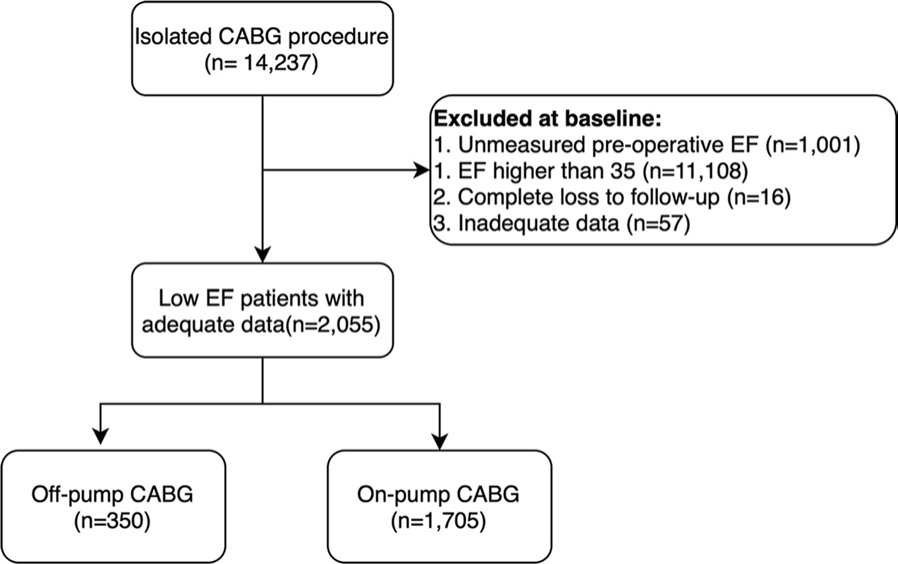
Study cohort flow-chart

Totally 14,237 patients underwent isolated CABG surgery between January 2014 and December 2016 were included in this study. After concerning inclusion and exclusion criteria 2055 patients were remained (Fig. 1). Consequently, 350 and 1705 patients underwent OPCABG and ONCABG, respectively. The median follow-up period was 47.83 [38.41, 55.19] months.

Patients’ demographics and preoperative data are summarized in Table 1. Briefly, 24% and 19.7% of patients were female in OPCABG and ONCABG (*p* = 0.070), respectively. The mean age of individuals was 62.05 years in OPCABG and 61.82 years in ONCABG (*p* = 0.429).

**Table 1:** Patients’ baseline characteristics

	Off-pump (n = 350)	On-pump (n = 1705)	*P* value
Age (years)	62.05 ± 0.54	61.82 ± 0.24	0.429
Female	24% (84)	19.7% (336)	0.070
BMI > 30 (kg/m^2^)	20.6% (72)	17.8% (304)	0.227
Graft number	3 [3, 4]	4 [4, 5]	< 0.001
EF (%)	29.1 ± 0.29	30.9 ± 0.11	< 0.001
Creatinine (mg/dl)	0.96 [0.93, 1.01]	0.96 [0.94, 1.00]	0.798
*eGFR (ml/min)*			
> 90	34.3% (120)	34.8% (594)	0.411
90–60	42.9% (150)	39.5% (673)	
< 60	22.9% (80)	25.7% (438)	
Positive family history	22.6% (79)	28.3% (482)	0.029
Diabetes mellitus	48.6% (170)	45.0% (767)	0.220
Hypertension	55.4% (194)	49.1% (838)	0.032
Dyslipidemia	52.0% (182)	45.7% (780)	0.033
Renal Failure	5.8% (20)	4.2% (71)	0.192
COPD	4.9% (17)	5.1% (87)	0.849
Cardiovascular accident	13.1% (46)	9.9% (169)	0.072
Opium user	20.9% (73)	21.7% (370)	0.727
Current cigarette smoker	21.4% (75)	23.6% (402)	0.386
Pre-surgery PCI	15.1% (53)	9.7% (165)	0.002
LM stenosis > 50%	11.7% (41)	11.3% (197)	0.832
No history	53.7% (188)	47.4% (808)	0.123
*Previous myocardial infarction*			
≤ 7 days	13.1% (46)	13.6% (232)	
8–21 days	8.6% (30)	11.7% (199)	
> 21 days	24.6% (86)	27.3% (466)	
Urgent/emergent procedure	0.6% (2)	1.8% (30)	0.102

*BMI* body mass index, *Hb* hemoglobin, *eGFR* estimated glomerular filtration rate, *EF* ejection fraction, *PCI* percutaneous coronary intervention, *LM* left main artery, *COPD* chronic obstructive pulmonary disease

### Survival outcomes

Table 2 demonstrated the absolute number of 4-P MACE events. In-hospital and mid-term mortality was significantly lower in OPCABG group, (2.0% vs 4.0%, *p* < 0.001, and 19.1% vs 26.4%, *p* < 0.001). Hence; the incidence of ACS and revascularization were same in both technique.

**Table 2:** Incidence rate in each group

	Off-pump (n = 350)	On-pump (n = 1705)	*P*-value
In-hospital mortality	9 (2.0%)	68 (4.0%)	< 0.001
Mid-term mortality	67 (19.1%)	450 (26.4%)	< 0.001
ACS	15 (4.3%)	59 (3.5%)	0.121
CVA	9 (2.6%)	47 (2.8%)	0.451
Revascularization	1 (0.3%)	9 (0.5%)	0.742

### First 30-days mortality

In-hospital mortality was lower in OPCABG group (OR: 0.136; 95%CI [0.068, 0.274], *p* < 0.001) in non-adjusted model and adjusted inverse probability weighting-based model (OR: 0.21; 95%CI [0.14, 0.52], *P* < 0.001).

### All-cause mortality

**Fig. 2:**
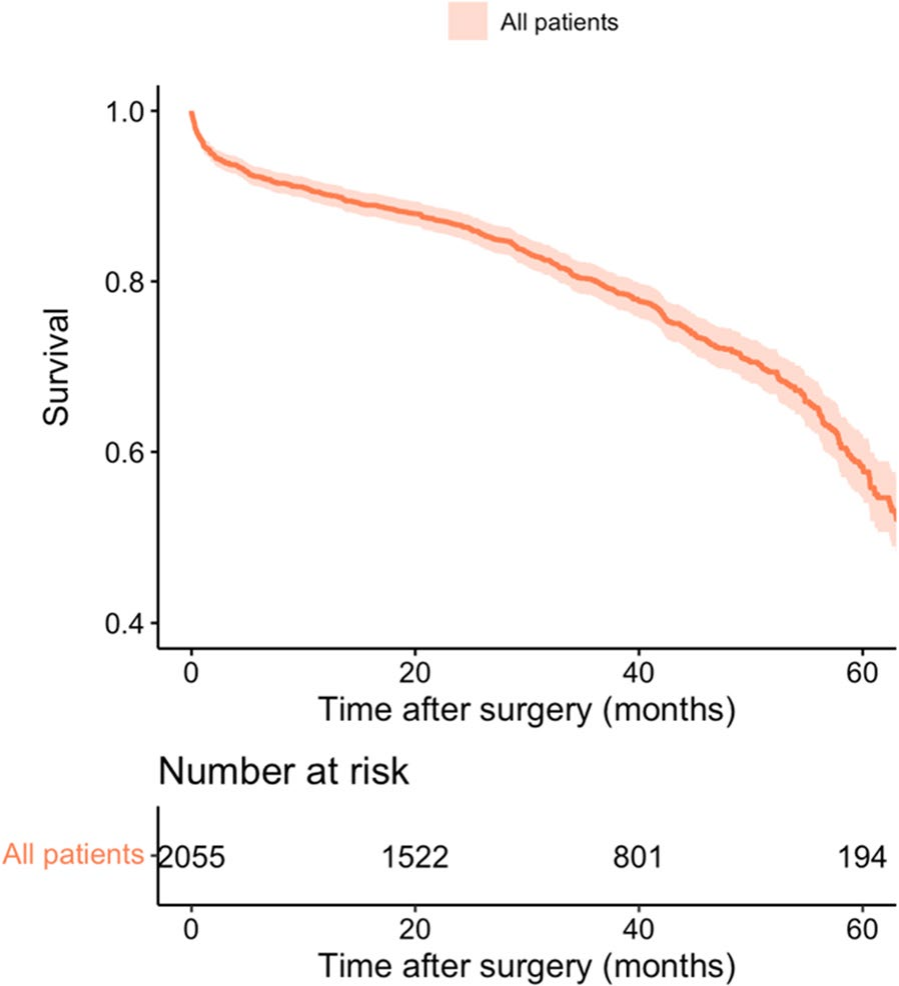
Mid term survival in patients with severe left ventricular dysfunction after CABG

**Fig. 3:**
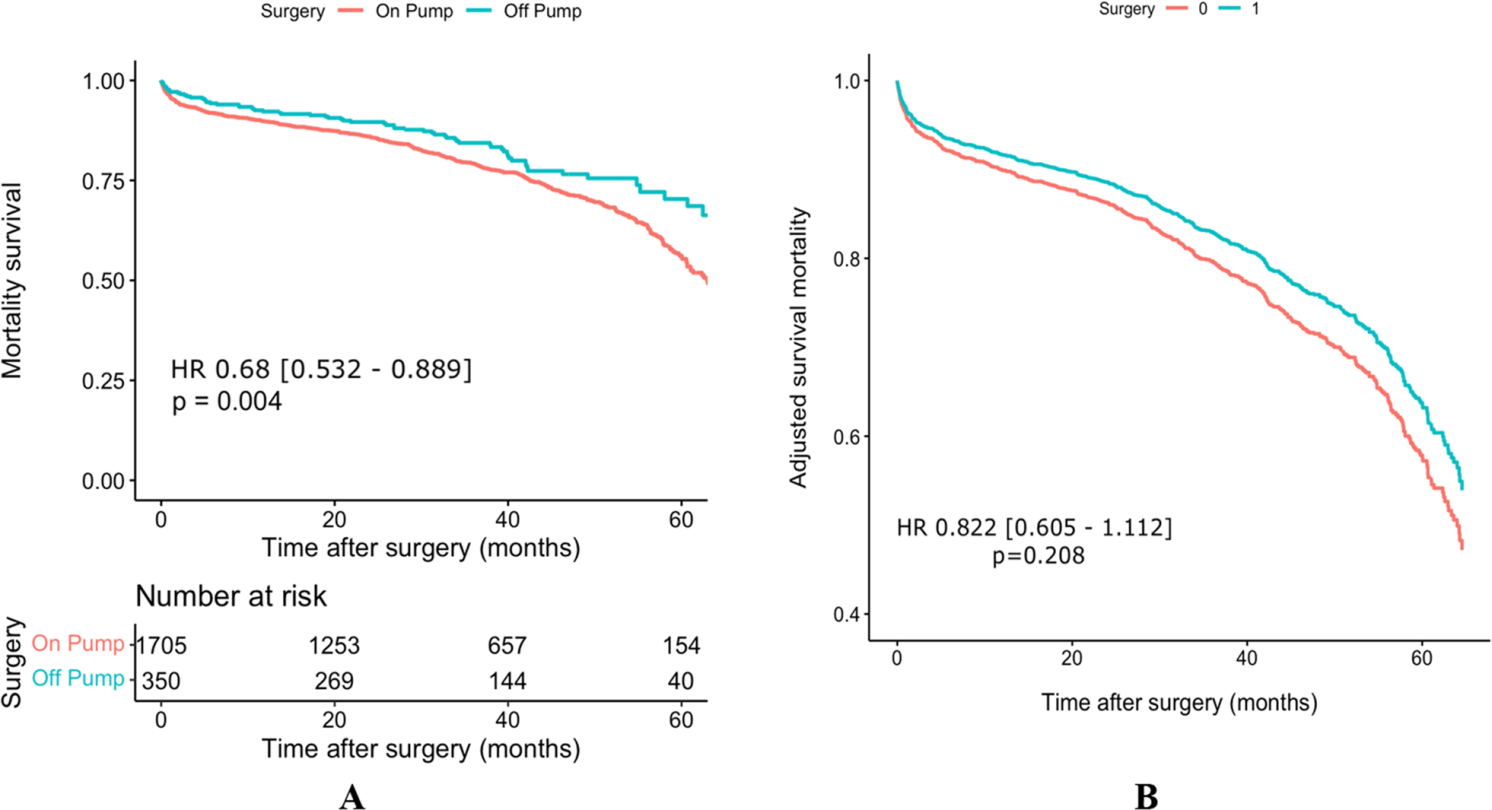
Unadjusted (**A**) and IPW adjusted (**B**) mortality survival in OPCABG and ONCABG groups

All-cause mortality rate was 25.16 percent in overall (Fig. 2). Table 2 demonstrated incidence rate in each studied group. OPCABG was associated with lower risk of mortality (HR: 0.687; 95%CI [0.532, 0.889], *p* = 0.004) (Fig. 3A). However, after adjustment for different confounders according to weighted method (IPW technique), there was no differences between two surgical methods (HR: 0.822; 95%CI [0.605, 1.112], *p* = 0.208) (Fig. 3B). Sensitivity analysis with in parallel with our main results (Table 3).

**Table 3:** Sensitivity analysis

All-cause mortality		Non-fatal CVEs	
HR	*P* value	HR	*P* value
0.812 [0.799–1.229]	0.823	1.423 [0.921–2.012]	0.231

### Non-fatal cardiovascular events (CVEs)

**Fig. 4:**
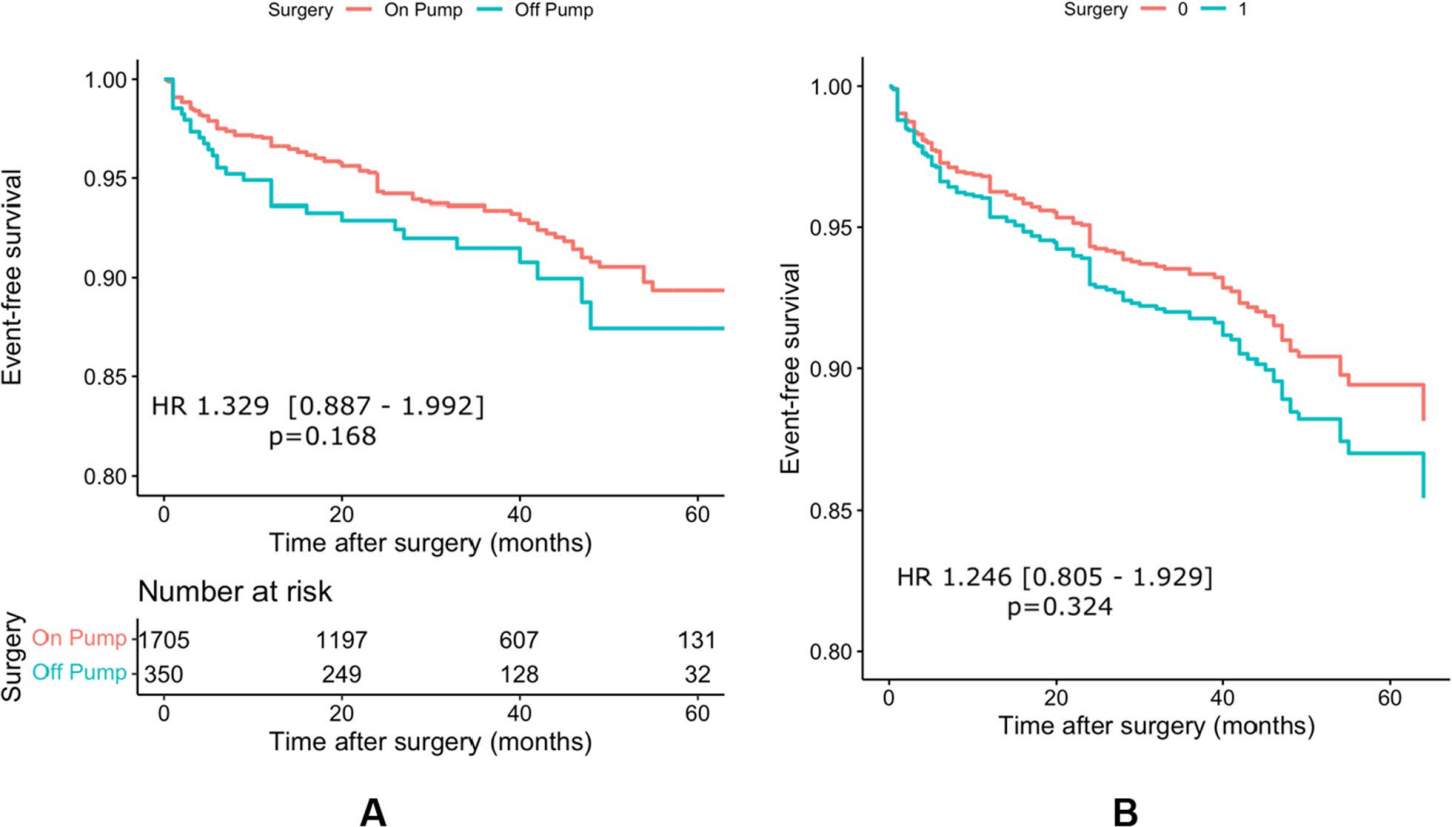
Undadjusted (**A**) and IPW adjusted (**B**) non-fatal CVEs survival in OPCABG and ONCABG groups

Non-fatal CVEs rate was 6.8 percent among all patients. In both non-adjusted and adjusted models, non-fatal CVEs were not different between two groups; HR: 1.329 95%CI [0.887, 1.992], *p* = 0.168 for non-adjusted model) (Fig. 4A) and (HR: 1.246; 95%CI [0.805, 1.929], *p* = 0.324) (Fig. 4B) in IPW.

### Subgroup Analysis

Table 4 demonstrate subgroup analysis for mortality and non-fatal CVEs. “Risk factor > 3” was the single factor which modify the results, which was not significant according to multiple test correction. There were no other factors which impact our main results.

**Table 4:** Subgroup analysis

	All-cause mortality		Non-fatal CVEs	
	HR	P* value	HR	P* value
More than 3 risk factors	0.420 [0.178–0.992]	0.048	1.208 [0.449–3.247]	0.708
Diabetes	0.805 [0.521–1.242]	0.327	0.888 [0.452–1.745]	0.730
Hypertension	0.789 [0.521–1.196]	0.264	1.118 [0.602–2.075]	0.725
Dyslipidemia	0.774 [0.506–1.184]	0.237	0.851 [0.444–1.631]	0.627
Age ≥ 70	0.939 [0.581–1.517]	0.797	1.375 [0.581–3.258]	0.469
Male	1.112 [0.610–2.102]	0.451	1.518 [0.643–3.582]	0.341
Female	0.712 [0.512–1.078]	0.098	1.094 [0.640–1.871]	0.742

^*^Significant *p*-value was considered as < 0.007

## Discussion

In this conducted study, we aimed to compare 30-day mortality, mid-term mortality and mid-term non-fatal CVEs in 2055 patients with left ventricular dysfunction (EF ≤ 35%) who underwent either off or on pump CABG procedure. In-hospital mortality was significantly lower in patients underwent OPCABG. However, mid-term results were comparable in both groups. In current study, we implanted IPW, which uses the whole data set and by reweighting individuals, increases the weight of those with unexpected exposures; ultimately, IPW creates a pseudo-population in which the covariates are balanced excellent between studied groups [17].

Management of patients with CAD and low EF remains a challenge; however, CABG seems to be a good surgical option in terms of survival and quality of life [18]. Moreover, ventricular dysfunction is considered as a risk factor for worse prognosis after cardiac surgery [19, 20]. The lower risk of in-hospital mortality in patients underwent OPCABG compared to ONCABG could be partly explain by the effect of CPB. Using CPB pump, as in ONCABG procedure, could increases organism’s inflammatory, oxidative and coagulators stress [21] mostly due to conversion of laminar flow, interaction of blood with the artificial bypass surface, hypothermia, and cold cardiac ischemia; consequently, this may affect outcomes of patients after CABG especially low EF population. Additionally, damaging effect of CPB could be partially explained by changing the geometry of left ventricle, which causes obstruction of coronary collateral flow that supplies ischemic myocardium [22]. Instead, OPCABG is associated with less inflammatory release, less hypercoagulable state, reduced transfusion requirement, and lower risk of postoperative kidney disease and may be more beneficial in patients with low EF [23, 24]. Based on previous conducted study, one possible reason for this improvement in in-hospital outcomes after OPCABG for patients with low EF may be the lack of ischemia during off-pump procedure and the performance of fewer distal anastomoses [25].

Based on previous studies, high-risk CABG candidates may benefit from avoiding CPB (OPCABG) [22, 26, 27]. This is in line with our findings which indicated that high-risk patients with more than three CAD risk factors had better survival when underwent OPCABG. This may be explained by CBP inflammatory reactions, higher risk of myocardial infarction after ONCABG [28], and renal dysfunction after ONCABG due to systemic inflammation and hypoperfusion [29]. It has been shown that oxidative metabolism recovers rapidly after off-pump bypass surgery and also the degree of myocyte injury and intraoperative cardiac troponin T concentrations are lesser in OPCABG compared to on-pump surgery [28]. Thus, elimination of CBP in addition to use of minimal incisions, as are seen in off-pump coronary bypass, may help to reduce the inflammatory reactions and lead to better outcomes compared to ONCABG [22, 30].

Although several studies have been compared off-pump and on-pump CABG in patients with low EF, the results are conflicting. Few studies showed that OPCABG was associated with lower risk of in-hospital mortality [2, 25], and some showed that in-hospital outcomes were the same between OPCABG and ONCABG [31–35]. Regarding mid- and long-term outcomes, in line with our results, some studies showed similar risk between off- and on-pump procedures [33, 34]. There are several conducted studies in this field, although none of them consider individuals genomic and epigenomic profiles composed. Future studies should identify the finest care for an individual based on a unique personal profile instead of the normal population [36].

The present study should be interpreted in the context of several possible limitations. First, due to lack of “cause of death” data recording, we were unable to specify “cause of death” in each patient. Moreover, unmeasured variables including surgery time duration and post-operative variables may alter our results and identified or unadjusted confounding effects cannot be ruled out for the association of lower 30-day mortality with OPCABG. Furthermore, our findings were based on 4-year follow up, and further studies with longer follow-up are needed to achieve results that are more accurate. This study was conducted in a single medical center (THC) and the generalizability of our results should be assessed. Still, THC is the referral educational university, which serves patients from all of the country. In order to precisely compare OPCABG and ONCABG we need large randomized clinical trials however, with IPW technique, we tried to overcome this limitation. IPW method balance two groups according to their risk factors, which computes from propensity scores for each patient.

The major strengths of this study are as follows; first, we adjusted our results with IPW to overcome the influence of baseline characteristics differences on the final result; hence, all study population maintained; second, our data extracted from THC registry data bank which records patient’s data prospectively; third, to overcome surgical expertise limitation, we chose expert surgeons who had done at least 100 and 400 OPCABG and ONCABG procedures previously.

In conclusion, for patients with ventricular dysfunction and EF ≤ 35% who need surgical coronary revascularization, OPCABG techniques compared to ONCABG strategies are associated with superior short-term results and comparable or even better mid-term outcomes, especially in those with multiple risk factors.

## Supplementary Information

**Additional file 1. Table S1**. Variables used in propensity score estimations.** Fig. S1**. C-statistic for propensity score modeling.** Fig. S2**. Estimated Propensity scores.** Table S2**. Standardized mean differences (SMD) percentage of characteristic variables.

## Data Availability

The datasets generated and analyzed during the current study are not publicly available due Tehran Heart Center data privacy protocol, but are available from the corresponding author (K.H) on reasonable request.
