# Supplementary material for: Off-pump versus on-pump coronary artery bypass graft surgery outcomes in patients with severe left ventricle dysfunction: inverse probability weighted study
Source: BMC Cardiovasc Disord. 2022 Nov 17;22:488. doi: 10.1186/s12872-022-02895-0 (PMC9673356; doi:10.1186/s12872-022-02895-0)
Supplement: Supplementary file 1 — Additional file 1. Table S1. Variables used in propensity score estimations. Fig. S1. C-statistic for propensity score modeling. Fig. S2. Estimated Propensity scores. Table S2. Standardized mean differences (SMD) percentage of characteristic variables. [file 12872_2022_2895_MOESM1_ESM.docx]

| Table S1  **Variables used in propensity score estimations** |
| --- |
| **Demographic**  Female  Age  BMI <30 and ≥30  Opium  Current cigarette smoker |
| **Medical history**  Diabetes  Hypertension  COPD  Cerebrovascular accident  Pre surgery PCI  Positive family history |
| **Preoperative lab test**  GFR |
| **Cardiac status**  Graft number  Ejection fraction  LM stenosis  Previous myocardial infarction (No history, ≤7 days, 8-21 days, **>21days)** |
| **Surgical decision**  Urgent operation |


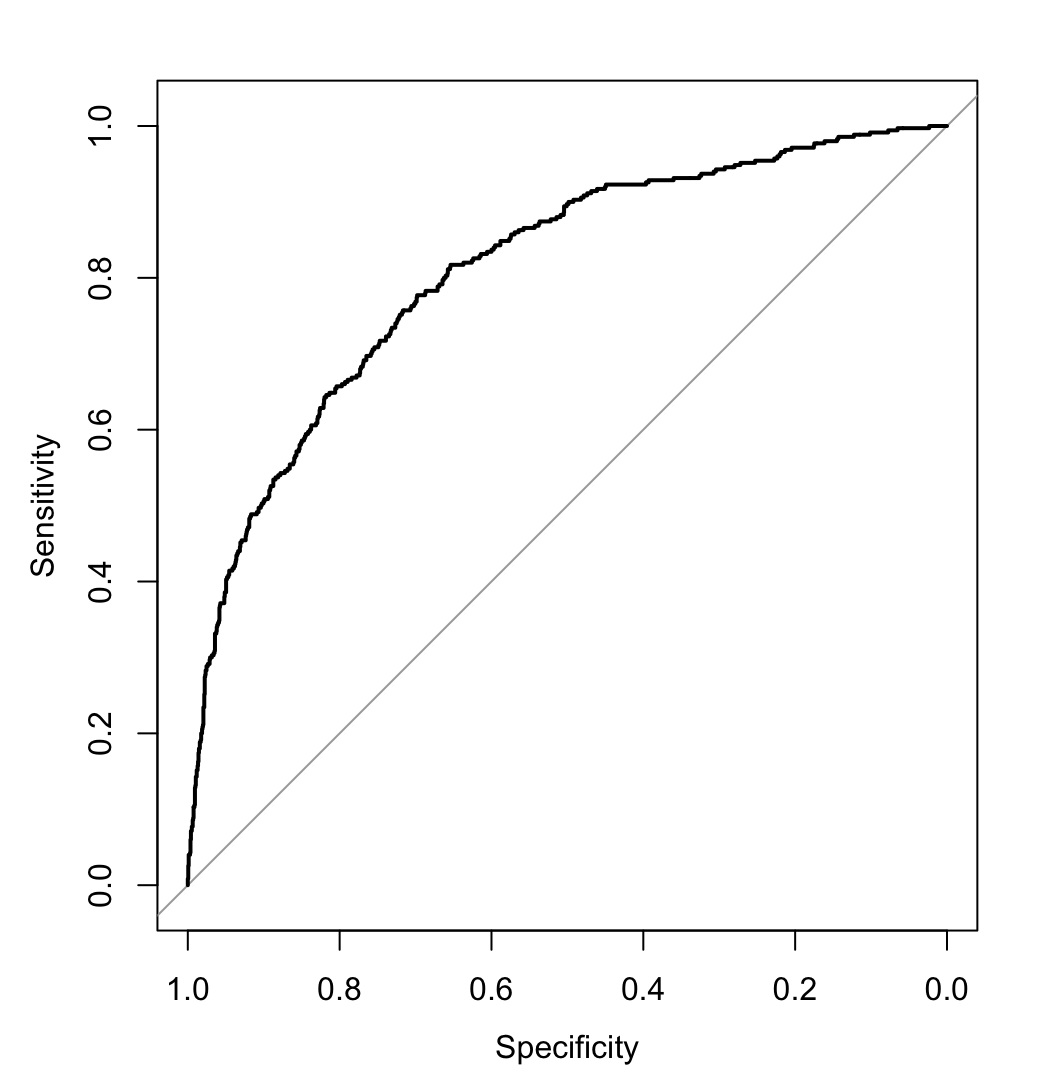


Fig. S1. C-statistic for propensity score modeling


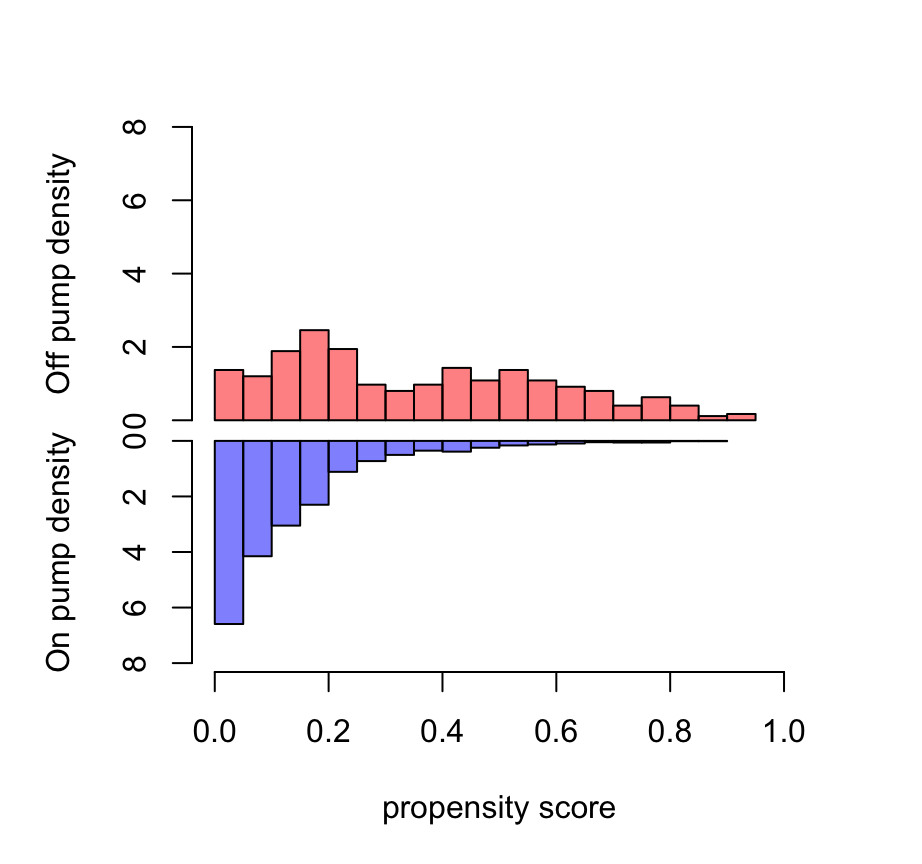


Fig. S2. Estimated Propensity scores

| Table S2. Standardized mean differences (SMD) percentage of characteristic variables | | |
| --- | --- | --- |
|  | Unadjusted | IPW |
| eGFR | 0.041 | 0.088 |
| Age | 0.148 | 0.055 |
| Gender | 0.015 | 0.015 |
| Dyslipidemia | 0.127 | 0.054 |
| Diabetes | 0.045 | 0.033 |
| Hypertension | 0.180 | 0.044 |
| Positive Family History | 0.049 | 0.012 |
| Opium | 0.051 | 0.044 |
| Current cigarette smoker | 0.077 | 0.011 |
| EF | 0.181 | 0.041 |
| Left main stenosis | 0.007 | 0.012 |
| Pre surgery PCI | 0.189 | 0.005 |
| BMI | 0.014 | 0.006 |
| Urgent operation | 0.031 | 0.120 |
| COPD | 0.081 | 0.038 |
| Cerebrovascular Accident | 0.018 | 0.037 |
| Previous myocardial infarction (≤7 days) | 0.109 | 0.012 |
| Previous myocardial infarction (8-21 days) | 0.122 | 0.075 |
| Previous myocardial infarction (>21days) | 0.011 | 0 |
| Graft number | 0.907 | 0.128 |
